# Surface Induced Order in Room Temperature Ionic Liquids and Its Effect on Piezoelectric Response

**DOI:** 10.1021/acsmaterialslett.5c01480

**Published:** 2025-12-22

**Authors:** Neelanjana Mukherjee, G. J. Blanchard

**Affiliations:** 3078 Michigan State University, Department of Chemistry, 578 S. Shaw Lane, East Lansing, Michigan 48824, United States

## Abstract

We compare the direct piezoelectric response of the room temperature ionic liquid (RTIL) *N*-butylpyridinium bis­(trifluoromethyl-sulfonyl)­imide (C_4_Py TFSI) under conditions where pressure is applied to the bulk RTIL in a vessel with a bare ITO interface and a vessel with an ITO interface modified with a monolayer of a pyridinium-containing amphiphile. We find that the presence of a single monolayer of the pyridinium amphiphile poises the RTIL structurally to produce a measurably larger piezoelectric response. The extent of order imposed by the presence of the monolayer is likely limited by the intrinsic surface roughness and structural irregularity of the ITO-coated glass support used.

## Graphical abstract

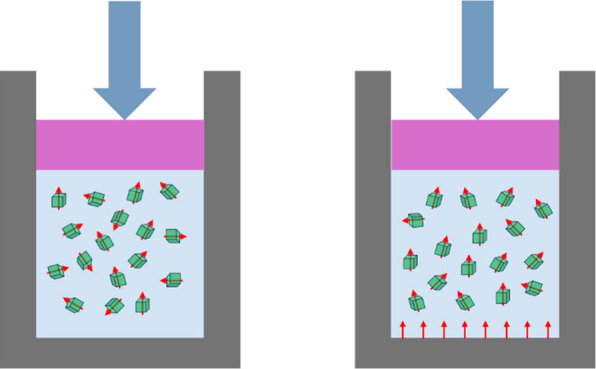

Room temperature ionic liquids (RTILs) have received extensive attention because of their demonstrated utility in fields ranging from double layer capacitors^1,2^ and ion propulsion^3–5^ to gas sequestration^6,7^ and use as an organic reaction medium.^8–10^ Despite the widespread use of RTILs, there is much that remains to be understood about the fundamental intermolecular interactions responsible for the useful properties. This limitation is driven by the high charge density that characterizes these systems as well as their high viscosity. A persistent theme that has emerged in the study of RTILs is their dynamical spatial heterogeneity, even at relatively high dilution.^11–13^ The inability to treat RTILs as homogeneous media as limited the ability to model them and at the same time, this heterogeneity is responsible for some of the useful applications.

We have recently reported that RTILs exhibit the direct piezoelectric effect.^14–16^ The application of pressure to the liquid phase RTIL gives rise to a liquid-to-crystalline solid phase transition, and the crystals formed do not possess a center of symmetry.^15^ This finding is the result of extensive investigation of an induced charge density gradient in RTILs that extends on the order of 50 μm into the bulk medium when placed in contact with a charged surface.^12,13,17–22^ This induced charge density gradient is the manifestation of the converse piezoelectric effect in these systems. An important question regarding the existence of the direct piezoelectric effect in RTILs is how the molecular structures of the RTIL constituents influence the magnitude of the piezoelectric response. This question bears not only on the extent to which charge can be distributed and displaced in the pressure-induced crystals, but also on the nature of the phase transition accessed by the application of pressure. Pressure-induced phase transitions have been reported for a number of RTILs,^23–34^ but there is a paucity of data in the pressure regime we access experimentally to observe the direct piezoelectric response. Confounding this matter further is the fact that certain RTILs can exhibit liquid-to-glass-to-crystal or liquid-to-crystal-to-glass phase transitions that depend on the rate and magnitude of pressure applied. Despite all these remaining structural and dynamical issues related to the piezoelectric response of RTILs, if the nominally amorphous liquid-to-solid phase transition could, in principle, be modified to poise the system to crystallize, then an expected consequence would be a change in the magnitude of the direct piezoelectric response. There is precedent in the materials community for using interfacial properties to poise a liquid phase system to adopt a favorable nascent orientation. Templated ordering is known to be effective in liquid crystal systems.^35–39^

We have deposited a monolayer of amphiphiles bearing a structural resemblance to the RTIL cationic pyridinium moiety on an ITO surface using Zr-bisphosphonate (ZP) chemistry^40–45^ and demonstrate that the presence of this monolayer has a subtle but measurable effect on the piezoelectric response of an RTIL in contact with the monolayer, upon the application of pressure. We observe that the presence of the monolayer gives rise to a change in the orientational distribution of the RTIL constituents such that a larger fraction of the pressure-induced crystals is aligned with the axis along which force is applied. We believe that, while subtle, this effect has important implications for optimizing the piezoelectric response in RTILs.

## Materials

Room-temperature ionic liquid *N*-butylpyridinium bis­(trifluoromethyl-sulfonyl)­imide (C_4_Py TFSI) (Figure 1) was purchased from Sigma-Aldrich and purified prior to use according to a procedure reported previously.^17,46^ Zirconyl chloride octahydrate (ZrOCl_2_·8H_2_O, 98%), anhydrous acetonitrile (CH_3_CN anhydrous, 99.8%), phosphorus­(V) oxychloride (POCl_3_, 99%), 2, 4, 6-trimethylpyridine (collidine, 99.8%), methanol (99.8%), isopropanol (>99.5%) and ethanol (>99.5%) were purchased from Sigma-Aldrich. (12-Dodecylphosphonic acid)­pyridinium chloride (>97%) was purchased from Sikemia. All reagents were used as received, without further purification. Ultrapure Milli-Q water (18 MΩ) was supplied by a Thermo Scientific Genpure system and used in all experiments. The ITO coated glass discs were purchased from Nanocs Inc. (IT10–111–25, 10 Ω sq^–1^).

**Figure 1:**
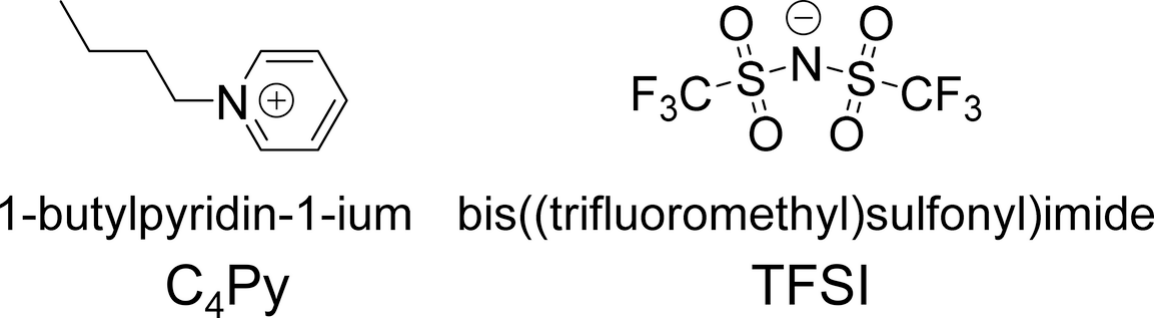
Chemical structure of the ionic liquid used.

## Surface Preparation

The ITO discs were cleaned by immersion in Milli-Q water and detergent (Fisher Sparklin 1) and sonicated for 15 min. The ITO-coated discs were rinsed with Milli-Q water to remove detergent, then immersed in Milli-Q water and sonicated for 15 min followed by isopropanol for 15 min. After rinsing with ethanol, the ITO supports were stored in Milli-Q water prior to use.

## Monolayer Deposition

The cleaned ITO discs (dried under N_2_) were phosphated directly using POCl_3_ and collidine in anhydrous acetonitrile in a fume hood. After 10 min, the substrates were rinsed with Milli-Q water and dried under a stream of N_2_. The discs were zirconated by immersion in a 5 mM solution of ZrOCl_2_ in ethanol (aqueous, 60% v/v) for 5 min. For the monolayer formation, the discs were immersed in a 1 mM solution of (12-dodecylphosphonic acid)­pyridinium chloride in methanol for 10 min. The ITO discs were stored in a KCl solution to anneal the system. The ITO discs were dried in the oven at ∼ 110 °C for 30 min before use (Figure 2).

**Figure 2:**
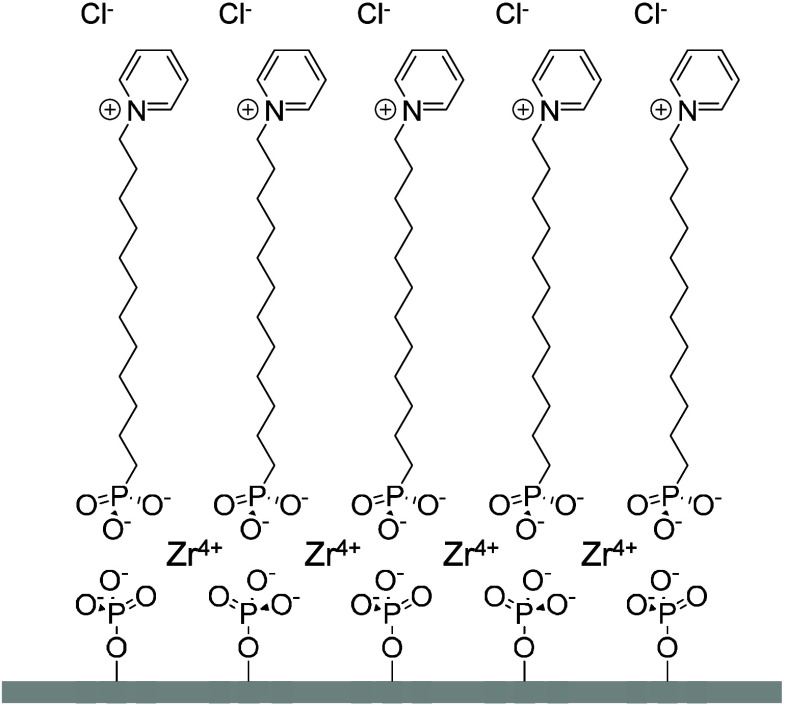
Schematic of monolayer formed on the ITO surface.

## Direct Piezoelectric Response Measurement

Measurement of the magnitude of the direct piezoelectric effect was performed using an instrument designed and constructed in-house and described in detail elsewhere.^16^ This instrument holds a cylinder and piston assembly containing the RTIL sample, where the cylinder is metal (stainless steel), and the piston is made of Delrin and contains a center metal electrode. The cylinder can be disassembled to allow for ITO-coated glass or modified ITO-coated glass discs to be inserted as the cylinder head. The bare ITO disc (reference) and the surface-modified ITO-coated disc (sample) are inserted and 200 μL of C_4_PyTFSI RTIL is placed in contact with the ITO surface. The seal between the cylinder (with the ITO disc(s)) and piston is made using a Buna-N O-ring, and care is required to ensure a seal that allows air but not the RTIL to escape upon the application of force.^14,47^

The device is a class two lever that allows access to a range of forces up to a factor of 10 in excess of that accessed in our original report (∼450 N).^14^ The current through the cell is measured as a function of applied force using an electrometer (Keithley 6517B). The electrometer is controlled, and data are acquired using a LabVIEW VI computer program written in-house. The force applied is measured using a calibrated digital force gauge (Nidec model FG-3009).

With the discovery of the direct piezoelectric effect in RTILs, there are several questions that are central to utilizing the finding. We focus in this work on understanding in detail the effect and the implications of inducing order in the crystallization process within the RTIL upon application of pressure. We understand that the imposition of nascent organization in the liquid phase can, in principle, give rise to either a smaller or larger piezoelectric response than that recovered from a nominally randomly oriented liquid, because the monolayer we have deposited on the modified ITO-glass support may poise the system in an orientation at an angle with respect to the force axis that may not be optimal. Any change in the piezoelectric response based on the monolayer formed on the ITO-coated support will reflect the monolayer’s role in narrowing the orientational distribution of crystals formed upon application of pressure.

For the data reported here, an electrometer is used to measure charge. In this configuration, we measure the current generated as a result of the force application-and-release cycle. The application of force to the RTIL drives a liquid-to-crystalline solid phase transition which creates nanocrystals from the bulk liquid RTIL, with the magnitude of the resulting current being proportional to the force applied. We show in Figure 3 the current vs force data for a clean ITO-coated glass surface (solid circles) and for a modified ITO-coated glass surface (open circles). The data are pooled from multiple experimental runs and, while they appear to differ little, there is a clear difference between the slopes of these data that is beyond the experimental uncertainty. For the clean ITO-coated glass surface, we recover a slope of 3.59 ± 0.14 (1σ) nA/N and for the surface-modified ITO-coated glass surface, we recover a slope of 4.20 ± 0.18 (1σ) nA/N. There is thus a measurable difference between the clean and modified surfaces, consistent with the monolayer deposited on ITO structurally poising the liquid phase RTIL prior to the application of force. We note that the y-intercepts for the data shown in Figure 3 are related to the conductivity of the RTIL under zero force. We consider next the information content of these findings.

**Figure 3:**
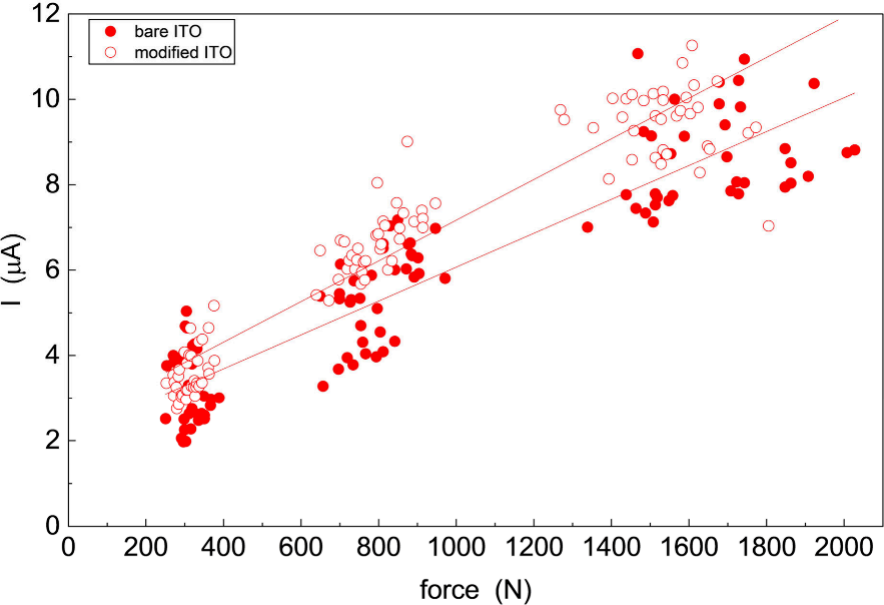
Relationship between transient current and force applied for C_4_Py TFSI on bare and modified ITO.

The interaction of a directional force upon a liquid with an orientational distribution of the pressure-induced crystals leads to the observed direct piezoelectric response, and for it to be seen, the force applied needs to align with the crystalline axis along which the charge separation occurs. The efficiency of coupling of the force applied to the piezoelectrically active crystal axis will scale with cos^2^θ, where θ is the angle between the force and active crystal axis. For a randomly distributed initial distribution of transient crystals, this distribution will appear as the black curve in Figure 4. We assert that the monolayer induces a narrower distribution of crystal orientations and assume that the total number of crystals formed is the same for both the clean and modified ITO interfaces. For a distribution of crystal orientations for the modified surfaces that scales with cosθ, the normalized distribution will appear as a cos^3^θ function (red curve in Figure 4 and for a) modified distribution that scales with cos^2^θ, we expect an overall cos^4^θ dependence (blue curve in Figure 4). The equations describing the normalized curves shown in Figure 4 are given by *f*_2_(θ), *f*_3_(θ) and *f*_4_(θ) (eq 1).

1 $$\begin{array}{cc} f_{1}\left(\theta \right)=\frac{1}{N_{1}}\int_{-\pi /2}^{\pi /2}\cos\left(\theta \right)dx & f_{2}\left(\theta \right)=\frac{1}{N_{2}}\int_{-\pi /2}^{\pi /2}\cos^{2}\left(\theta \right)dx\\ f_{3}\left(\theta \right)=\frac{1}{N_{3}}\int_{-\pi /2}^{\pi /2}\cos^{3}\left(\theta \right)dx & f_{4}\left(\theta \right)=\frac{1}{N_{4}}\int_{-\pi /2}^{\pi /2}\cos^{4}\left(\theta \right)dx \end{array}$$

where the terms *N*_*i*_ are the normalization factors for these equations. The recovered slopes of the clean and modified systems scale with the maximum normalized intensities (*I*_θ=0_) of the distribution associated with the modified and clean interfaces (eq 2).

2 $$\begin{array}{ccc} \frac{I_{\theta =0}\left(f_{2}\left(\theta \right)\right)}{I_{\theta =0}\left(f_{2}\left(\theta \right)\right)}=1 & \frac{I_{\theta =0}\left(f_{3}\left(\theta \right)\right)}{I_{\theta =0}\left(f_{2}\left(\theta \right)\right)}=1.178 & \frac{I_{\theta =0}\left(f_{4}\left(\theta \right)\right)}{I_{\theta =0}\left(f_{2}\left(\theta \right)\right)}=1.333 \end{array}$$

**Figure 4:**
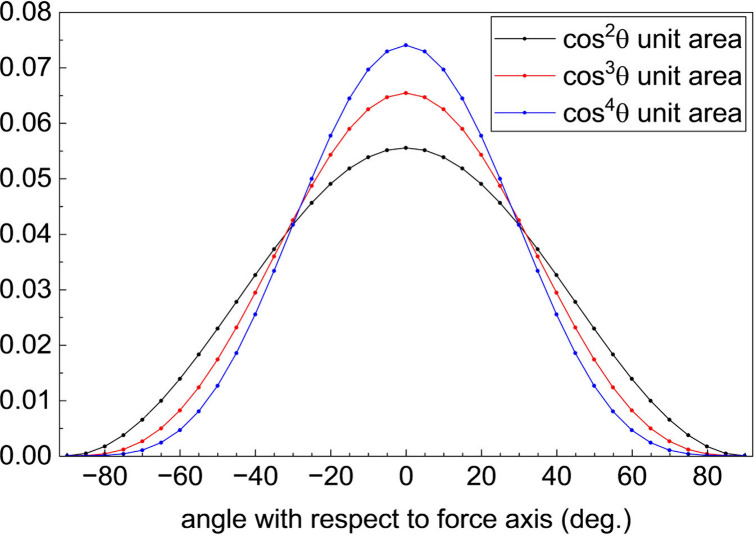
Normalized distribution functions induced by the surface modification.

If the deposited monolayer imparts no order, we expect the same result as for a clean ITO surface, with a slope ratio of 1. If the monolayer imparts order in the system that scales with cosθ, we expect a ratio of 1.178, and if the monolayer imparts order that scales with cos^2^θ, we expect a ratio of 1.333. Experimentally, the ratio of the slopes is seen to be

3 $$\frac{m_{\mathrm{modified}}}{m_{\mathrm{clean}}}=\frac{4.20\pm 0.18\;nA/N}{3.59\pm 0.14\;nA/N}=1.17\pm 0.07$$

The experimental data are consistent with the monolayer deposited onto the modified ITO surface imparting a cosθ orientational distribution of the transient crystals in the RTIL upon the application of force.

The functional form of the distribution invites consideration of its cause. Based on the use of an interfacial monolayer where the terminal functionality is very closely related to the identity of the RTIL cation (C_4_Py^+^), it is useful to consider the basis for the order imposed by the monolayer. Despite the structural match between the monolayer headgroup and C_4_Py^+^, there will not be direct interaction between these functionalities because of their charges. Rather, we expect that the surface modification will serve to present a more-or-less planar cationic interface to the RTIL, and for this configuration, the strongest interactions will be between the cationic monolayer and the RTIL anions. If the monolayer cationic interface were perfectly planar, it would poise pressure-induced crystal formation to have a very narrow orientational distribution which would appear to scale with cos^2^θ, producing a slope ratio of 1.333 (eq 2). We expect that the surface roughness and the intrinsically irregular distribution of reactive sites on the ITO surface leads to a significantly wider distribution of orientations at which the RTIL anions will interact with the monolayer, although experimental evaluation of the role of surface roughness is not feasible at present. In other words, the modified monolayer is not planar and the features that characterize the monolayer serve to increase the angular distribution of transient crystal orientations to produce a distribution intermediate between a random distribution and a single orientation, resulting in a distribution that will scale approximately as cosθ, in agreement with our observation.

We have reported for the first time the surface induced increase in the direct piezoelectric response in a bulk liquid-phase material, the room-temperature ionic liquid C_4_Py TFSI. The magnitude of the increase in the piezoelectric effect is an order of 17%. This change in the direct piezoelectric effect in RTILs implies order being induced in these media and these results point the way toward further experiments to further optimize the effect. A key issue to address in future work will be determination of the most influential structural, dipolar and/or electrostatic factors that can be controlled through surface-modification. The ability to change the piezoelectric response in a neat liquid opens the door to applications that have previously not been accessible with solid-state materials, and RTILs are more readily recyclable and in many instances pose fewer environmental issues than many currently used (solid state) piezoelectric materials.

## Data Availability

All data shall be made available upon request.
